# Use of Fermented Hemp, Chickpea and Milling By-Products to Improve the Nutritional Value of Semolina Pasta

**DOI:** 10.3390/foods8120604

**Published:** 2019-11-22

**Authors:** Rosa Schettino, Erica Pontonio, Carlo Giuseppe Rizzello

**Affiliations:** Department of Soil, Plant and Food Science, University of Bari Aldo Moro, 70126 Bari, Italy; rosa.schettino@libero.it (R.S.); carlogiuseppe.rizzello@uniba.it (C.G.R.)

**Keywords:** hemp, chickpea, milling by-products, fortified pasta, lactic acid bacteria, nutritional value

## Abstract

A biotechnological approach including enzymatic treatment (protease and xylanase) and lactic acid bacteria fermentation has been evaluated to enhance the nutritional value of semolina pasta enriched with hemp, chickpea and milling by-products. The intense (up to circa, (ca.) 70%) decrease in the peptide profile area and (up to two-fold) increase in total free amino acids, compared to the untreated raw materials, highlighted the potential of lactic acid bacteria to positively affect their in vitro protein digestibility. Fermented and unfermented ingredients have been characterized and used to fortify pasta made under pilot-plant scale. Due to the high contents of protein (ca. 13%) and fiber (ca. 6%) and according to the Regulation of the European Community (EC) No. 1924/2006 fortified pasta can be labelled as a “source of fiber” and a “source of protein”. The use of non-wheat flours increased the content of anti-nutritional factors as compared to the control pasta. Nevertheless, fermentation with lactic acid bacteria led to significant decreases in condensed tannins (ca. 50%), phytic acid and raffinose (ca. ten-fold) contents as compared to the unfermented pasta. Moreover, total free amino acids and in vitro protein digestibility values were 60% and 70%, respectively, higher than pasta made only with semolina. Sensory analysis highlighted a strong effect of the fortification on the sensory profile of pasta.

## 1. Introduction

The trend in world population growth (up to 9.6 billion people by 2050) and the necessity to provide a nutritionally balanced diet and to reduce greenhouse gas emissions require relevant production increases in vegetables, as well as a transition to a diet higher in plant- rather than animal-derived proteins [1,2]. Aiming at addressing environmental concerns and meeting nutritional deficiencies and recommendations, the fortification of staple foods (e.g., bread and pasta) has been identified as an effective, sustainable and promising intervention [3,4]. To date, several studies investigated the nutritional value of additional ingredients to be used as wheat alternatives in cereal-based products [4,5,6,7,8]. Due to its popularity [9], pasta has been proposed as a suitable carrier of nutrients, mainly dietary fiber and proteins [4,7,10,11,12,13].

Legumes are excellent sources of proteins with high biological value and dietary fibers and they supply high levels of vitamins, minerals, oligosaccharides and phenolic compounds [14]. Moreover, thanks to their functionality (e.g., solubility and water-binding capacity), legume flours have successfully been proposed to enhance gluten-free food formulation and processing [5].

Grain germ and bran (milling by-products) comprise important sources of dietary fiber and α-tocopherol, vitamins of group B, polyunsaturated fats, minerals and different bioactive compounds with health-promoting effects [15]. Pearling by-products, thanks to the high content of dietary fiber and β-glucan, have been suggested as suitable ingredients to produce functional pasta [6]. Hemp has recently raised much interest as a sustainable food ingredient due to the high content (ca. 30%) and biological value of protein, dietary fiber content (ca. 50%) as well as the considerable content of functional compounds (e.g., phenols) with antioxidant and anti-hypertensive properties [16].

Nevertheless, the poor stability to oxidation [17], the high content of fibers and the absence of gluten may impair the wheat alternative’s high nutritional value, worsening the technological and sensory profiles of the products [18]. Moreover, the presence of anti-nutritional factors (ANFs), i.e., phytic acid, condensed tannins, raffinose and trypsin inhibitors, further limit the use of such ingredients by the food industry [19]. Although different biotechnological options were suggested to overcome the drawbacks related to the use of non-wheat flours in cereal-derived foods, fermentation with lactic acid bacteria (LAB) seemed to be the best option to both decrease the ANF and improve their nutritional, technological and sensory profile [19,20].

Based on the above consideration, here, hemp and chickpea flours, and wheat milling by-products were proposed as additional ingredients to improve the nutritional quality of semolina pasta. Enzymatic pre-treatments and fermentation with LAB were evaluated as bioprocessing options to enhance protein digestibility and decrease ANF. Bioprocessed ingredients were used to manufacture fortified pasta, and the effects on the nutritional, technological, and sensory properties were investigated in comparison to samples produced with the untreated native ingredients.

## 2. Materials and Methods

### 2.1. Raw Materials, Bacterial Strains and Enzymes

Commercial hemp (*Cannabis sativa L*.) flour (Sottolestelle, San Giovanni Rotondo, Italy), chickpea grains (*Cicer arietinum L.* var. Pascià, Caporal Grani S.a.s.,Gravina di Puglia, Italy), wheat germ (Molino Rieper, Vandoies di Sotto, Italy) and bran (Molino Careccia, Stigliano, Italy) were used in this study. Chickpea, germ and bran flours were milled using a laboratory mill Ika-Werke M20 (GMBH, and Co. KG, Staufen, Germany) before use. After milling, all the flours were sieved (mesh size 500 µm) to remove the coarse fraction.

*Lactobacillus plantarum* LB1 and *Lactobacillus rossiae* LB5 [21] were used in this study. Strains were routinely cultivated on modified de Man Rogosa and Sharp (Oxoid, Basingstoke, Hampshire, UK) (mMRS) as reported by Rizzello et al. [21]. A commercial xylanase (880 *xylanase* u/g; Depol 761P, Biocatalysts Limited, Chicago, USA) from *Bacillus subtilis* and proteases of *Aspergillus oryzae* (500,000 hemoglobin units on the tyrosine basis/g; enzyme 1 [E1]) and *Aspergillus niger* (3000 spectrophotometric acid protease units/g; enzyme 2 [E2]), routinely used for bakery applications, supplied by BIO-CAT Inc. (Troy, VA, USA), were also used.

### 2.2. Proximate Chemical Composition of Raw Materials

Moisture, protein, lipids, total dietary fiber and ash of raw material were determined according to Approved Methods 44-15A, 46-11A, 30-10.01, 32-05.01, and 08-01.01 of the American Association of Cereal Chemists (AACC) [22]. Total nitrogen was corrected by 6.25, 4.99, 6.31 and 5.30 for chickpea, wheat germ, wheat bran and hemp, respectively [23,24]. Available carbohydrates were calculated as the difference [100 − (proteins + lipids + ash + total dietary fiber)]. Proteins, lipids, carbohydrates, total dietary fiber and ash were expressed as % of dry matter (d.m.).

### 2.3. Bioprocessing

Wheat germ and bran were mixed (1:4, *wt*/*wt*) prior to dough preparation. Doughs (50 g) were prepared by mixing hemp flour (62.5% *wt*/*wt*), or chickpea flour (62.5% *wt*/*wt*) or milling by-products (40.0% *w*/*w*) with tap water. Dough yield (DY, dough weight × 100/flour weight) was 160 for hemp (H) and chickpea (C), and 250 for milling by-products (WGB). To be used as a mixed starter for sourdough fermentation, LAB cells were harvested by centrifugation (10,000× *g*, 10 min, 4 °C), washed twice in 50 mM phosphate buffer, pH 7.0, and re-suspended in tap water used for the dough making (final cell density in the dough was ca. 7.0 log10 cfu/g). Fermentation was carried out at 30 °C for 24 h (H_F_, C_F_, and WGB_F_).

For the enzymatic treatments, doughs with the same DY were prepared. Before mixing, xylanase was added at 50 ppm based on dough weight (H_X_, C_X_, and WGB_X_) and proteases E1 and E2 (H_P_, C_P_, and WGB_P_) were used at 50 ppm (25 ppm for each enzyme) on dough weight. Doughs were incubated at 30 °C for 8 h. All the doughs were prepared and incubated in triplicate.

### 2.4. Determination of the Protein Degradation

Aiming at selecting the optimal bioprocessing option, total free amino acid (TFAA) concentration and peptide profiles were considered as indexes of the proteolytic degradation and screening parameters. Doughs prior bioprocessing were used as the controls (H, C, and WGB).

Water/salt-soluble extracts (WSEs) from doughs were prepared as reported by Weiss et al. [25] and used for TFAA and peptides analyses. TFAA were analyzed by a Biochrom 30 series Amino Acid Analyzer (Biochrom Ltd., Cambridge Science Park, United Kingdom) with a Na-cation-exchange column (20 by 0.46 cm internal diameter), as described by Rizzello et al. [17]. For the analysis of peptides, WSE were treated with trifluoroacetic acid (TFA, 0.05% *wt*/*v*) and subjected to dialysis (cut-off 500 Da) to remove proteins and FAA, respectively. Then, the peptide concentration was determined by the o-phtaldialdehyde (OPA) method [26]. Peptide profiles were obtained by Reversed-Phase Fast Performance Liquid Chromatography (RP-FPLC) [26,27], using an ÄKTA FPLC equipped with a Resource RPC column and a UV detector (214 nm) (GE Healthcare Bio-Sciences AB, Uppsala, Sweden). Peptide profiles and total peak area were elaborated with Unicorn 4.0 software (GE Life Sciences).

### 2.5. Microbiological and Biochemical Characterization of Fermented Doughs

pH values of H, C and WGB and H_F_, C_F_ and WGB_F_ were determined online by a pH meter (Model 507, Crison, Milan, Italy) with a food penetration probe. The AACC method 02-31.01 was used for the determination of total titratable acidity (TTA) of samples. Presumptive LAB was enumerated using de Man, Rogosa and Sharpe (MRS, Oxoid) agar medium, supplemented with cycloheximide (0.1 g/L). Plates were incubated, under anaerobiosis (AnaeroGen and AnaeroJar, Oxoid), at 30 °C for 48 h. WSEs from unfermented and fermented doughs were used for the determination of organic acids, peptides and TFAA concentrations. High-Performance Liquid Chromatography (HPLC) ÄKTA Purifier system (GE Healthcare, Buckinghamshire, United Kingdom) equipped with an Aminex HPX-87H column (ion exclusion, Bio-Rad, Richmond, CA, United States) and a UV detector operating at 210 nm [17] was used to quantify organic acids. The fermentation quotient (FQ) was determined as the molar ratio between lactic and acetic acids. Peptides and TFAA concentrations were determined as described above.

### 2.6. Pasta Making

Pasta was manufactured using a pilot plant La Parmigiana SG30 (Manufacture, Fidenza, Italy). Table 1 summarizes the ingredients and protocol used for pasta making. All the doughs had a final DY of 130, corresponding to 23% (*wt*/*wt*) water and 77% (*wt*/*wt*) dry matter (semolina, hemp, chickpea flours and milling by-products).

Unfermented and fermented doughs were obtained as described before and used as ingredients for pasta making. Due to the difference in terms of the DY (160 vs. 250) of the doughs, H, H_F_, C and C_F_ were used at 11% (*wt*/*wt*), while the level of fortification of WGB and WGB_F_ was 17% (*wt*/*wt*). Unfermented and fermented doughs were mixed with durum wheat semolina and water to obtain pasta samples (*p*H, *p*H_F_, *p*C and *p*C_F_ and *p*WGB and *p*WGB_F_, respectively). A control pasta was made without fortification (*p*CT). The process is composed of four stages: i) three-steps mixing (1 min mixing/6 min hydration); ii) extrusion of final dough at 45–50 °C through a n. 76 (150 mm diameter) bronze die; iii) cutting the extruded to obtain grooved “macaroni”; and iv) drying using low temperature (55 °C) cycle (Supplementary Table S1). The proximate composition of wheat semolina was moisture, 10.2%; protein, 12.1% of d.m.; fat, 1.8% of d.m.; ash, 0.6% of. d.m.; and total carbohydrates, 75.5% of d.m.

### 2.7. In vitro Protein Digestibility (IVPD)

The IVPD of pasta samples was determined according to Akeson and Stahmann [28,29]. In order to mimic the in vivo digestion in the gastrointestinal tract, pasta samples were subjected to a sequential enzymatic treatment. The IVPD is the percentage of the total protein solubilized after enzymatic hydrolysis. The protein quantification was made according to the Bradford method [30].

### 2.8. Pasta Characterization

#### 2.8.1. Hydration Test, Cooking Time, Cooking Loss and Water Absorption

The method of Marti et al. [31] (ratio pasta:water of 1:20, 180 min of incubation) was used to determine the hydration at 25 °C, while the method of Schoenlecher et al. [32] was used to determine the cooking time. The optimal cooking time (OCT) corresponded to the disappearance of the white core. Cooking loss (expressed as grams of matter loss/100 g of pasta) was evaluated by determining the number of solids lost into the cooking water as proposed by D’egidio, et al. [33]. The increase in pasta weight during cooking (water absorption) was evaluated by weighing pasta before and after cooking. The results were expressed as [(W1 − W0/W0] × 100, where W1 is the weight of cooked pasta and W0 is the weight of the uncooked samples.

#### 2.8.2. Chemical and Nutritional Profile

Chemical characteristics (determined on pasta dough prior to extrusion) and the proximal composition of pasta were determined as reported above.

The protein solubility of pasta (grinded) was evaluated under native and denaturing conditions as reported by Iametti et al. [34]. The concentration of protein and peptides was determined as reported above [7,30].

Raffinose and phytic acid concentrations were determined by using the Megazyme kit Raffinose/D-Galactose Assay Kit K-RAFGA and K-PHYT 05/07 (Megazyme International Ireland Limited, Bray, Ireland), respectively, following the manufacturer’s instructions. Condensed tannins were determined using the acid butanol assay, as described by Hagerman [35].

IVDP and starch hydrolysis were determined on pasta samples at the OCT. IVPD was determined as described before. The evaluation of the starch hydrolysis rate was performed using a procedure mimicking the in vivo digestion of starch [36]. Wheat flour bread (WB) was used as the control to estimate the hydrolysis index (HI = 100). The predicted GI of all pasta samples was calculated using the following equation: *p*GI = 0.549 × HI + 39.71 [37].

#### 2.8.3. Texture and Color Analysis

Instrumental Texture Profile Analysis (TPA) was carried out with a TVT-300XP Texture Analyzer (TexVol Instruments, Viken, Sweden), equipped with a cylinder probe (diameter 95 mm). For the analysis, pasta samples were cooked until the OCT, left to cool at room temperature and placed in a beaker (diameter, 100 mm; height 90 mm), filled to about half volume. The selected settings were the following: test speed 1 mm/s, 30% deformation of the sample and two compression cycles. The chromaticity coordinates of the samples (obtained by a Minolta CR-10 camera) were reported in the form of a color difference, ΔE × ab [7].

#### 2.8.4. Sensory Analysis

A trained sensory panel (*n* = 13, aged 21–45 years) assessed the sensory profile of pasta samples.

The lexicon consisted of twelve attributes as reported in Supplementary Table S2. A line scale from “not at all” (0) to “very” (10) for each attribute was used for the evaluation. Each pasta sample was cooked according to its own OCT and presented randomized in duplicate. Tap water was used to rinse the mouth between the samples. The study protocol followed the ethical guidelines of the sensory laboratory. A written informed consent was obtained from each participant.

### 2.9. Statistical Analysis

All analysis as well as the fermentation and enzymatic treatments were carried out in triplicate. The one-way ANOVA, using Tukey’s procedure at *p* < 0.05, was performed for the data elaboration (Statistica 12.5, StatSoft Inc., Tulsa, USA). Principal component analysis (PCA) with varimax rotation was performed to visualize the sensory characteristics of the samples with Unscrambler X10.3 (Camo SA, Trondheim, Norway).

## 3. Results

### 3.1. Proximate Composition of the Raw Materials

The proximate composition of the flours is reported in Table 2. Hemp flour was characterized by the highest concentration of protein (ca. 37% of d.m.) and total dietary fiber (ca. 39.7% of d.m.), while chickpea flour was characterized by the lowest concentration of fat (ca. 4% of d.m.) (Table 2).

### 3.2. Proteolysis and Set-Up of the Bioprocessing

**Figure 1:**
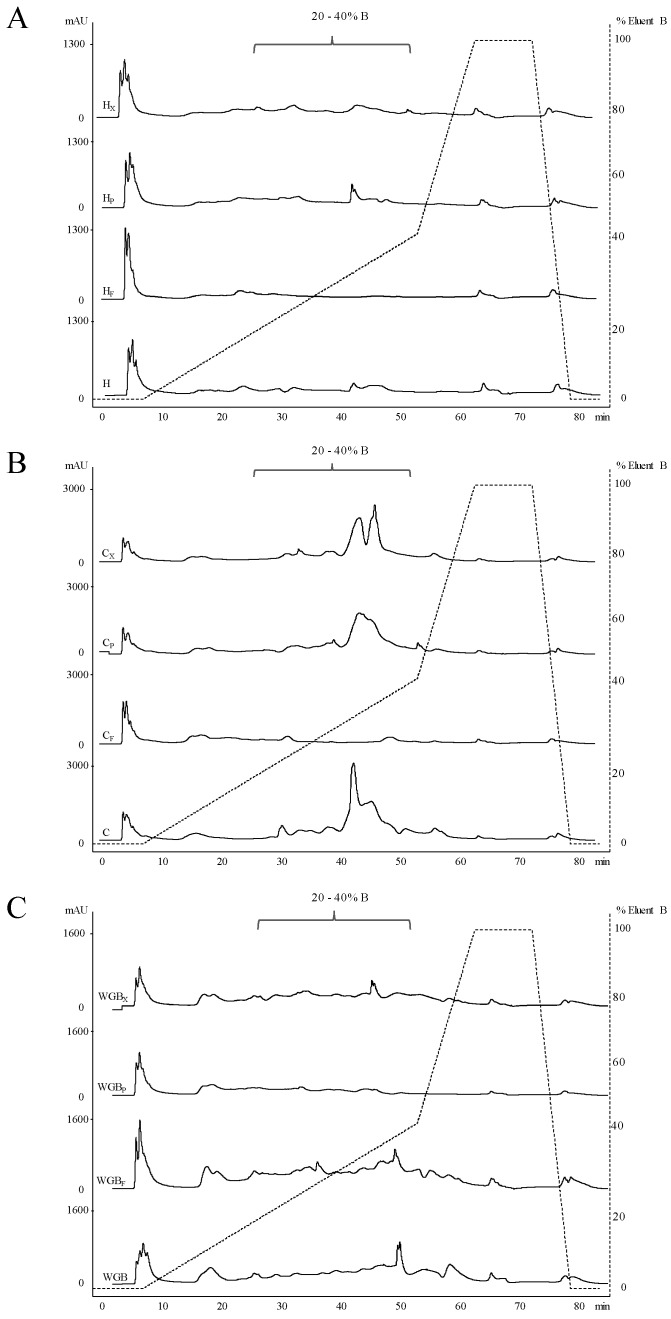
Reversed-Phase Fast Performance Liquid Chromatography (RP-FPLC) peptide profiles of hemp, chickpea and wheat germ/bran doughs. (**A**): H, untreated hemp dough; H_F_, fermented hemp dough; H_P_, hemp dough treated with proteases (E1/E2); H_X_, hemp dough treated with xylanase (Depol 761P). (**B**): C, untreated chickpea dough; C_F_, fermented chickpea dough; C_P_, chickpea dough treated with proteases (E1/E2); C_X_, chickpea dough treated with xylanase (Depol 761P). (**C**): WGB, untreated wheat germ/bran (1:4) dough; WGB_F_, fermented wheat germ/bran (1:4) dough; WGB_P_, wheat germ/bran (1:4) dough treated with proteases (E1/E2); WGB_X_, wheat germ/bran (1:4) dough treated with xylanase (Depol 761P).

TFAA and peptide profiles were used as screening criteria for bioprocessing parameters, since they correspond to the organic nitrogen compounds released during the process from native proteins. According to the peptide profiles (Figure 1), H and WGB were characterized by a total peak area significantly lower than C (3479 ± 34 and 5194 ± 25 mAU × mL vs. 15011 ± 53 mAU × mL). As the consequence of the bioprocessing, changes were observed mainly in the range 20% to 40% of the acetonitrile gradient, while the hydrophilic zone had undergone minimal alterations (Figure 1). Enzymatic treatments led to slight increases in the total peak area of peptides. Values from 6.8% (C_P_) to 9.4% (WGB_P_) higher were found when proteases were used. Similarly, increases up to ca. 10% were found in C_X_ and WGB_X._ H_X_ and H were characterized by a similar peptide area. On the contrary, LAB fermentation caused a significant decrease in the peptide profile area, with H_F_ and C_F_ characterized by a relevant lower area (21 and 71%, respectively) than H and C.

The concentration of TFAA in H, C and WGB prior to bioprocessing ranged from 802 ± 5 to 1972 ± 12 mg/Kg (Table 3). LAB and enzymes both increased TFAA concentration. Among the enzymatic treatments, proteases led to a concentration from ca. 3% (C_P_) to 70% (H_P_) higher than the corresponding untreated controls, while increases in the range 2 (C_X_) to 40% (WGB_X_) were found when xylanase was used. LAB led to the highest increases in the TFAA concentration (up to two-fold higher) (Table 3). In detail, the highest TFAA concentration was found in C_F_ (2457 ± 16 mg/Kg).

Although both enzymatic treatments caused a moderate increase in the peptides in treated samples, the most extensive protein degradation that occurred during fermentation suggested a more intense potential effect of the LAB on protein digestibility. According to these considerations, fermented samples were subjected to further analysis.

### 3.3. Chemical Characterization of Doughs

A decrease in pH of ca. 2 units was achieved in all fermented doughs (Table 3). Significant higher values of TTA were found in H_F_, C_F_ and WGB_F_ compared to H, C and WGB, respectively. These changes are in accordance to the increases in lactic and acetic acids in fermented doughs. The highest concentration of lactic acid was found in WGB_F_, while H_F_ contained the highest amount of acetic acid (Table 3). Decreases in the peptide concentrations (up to ca. 80%) were found after fermentation. The highest decrease was found in C_F_, while, according to the TFAA concentration, the lowest value was found in H_F_ (Table 3).

### 3.4. Chemical, Technological and Structural Properties of Pasta

The inclusion of both unfermented and fermented ingredients affected the chemical characteristics of pasta. However, the pH and TTA values differ from *p*CT only when fermented ingredients were used (Table 4). TFAA concentration was higher in all fortified pasta, as compared to the pCT with higher extent when fermented ingredients were used. *p*WGB_F_ contained the highest amount (ca. ten-fold higher than *p*CT) (Table 4).

The experimental OCT of *p*CT was ca. 10 min. Decreases (from 25 to 66%) in OCT were found for fortified pasta, especially when fermented ingredients were used (Table 4). A similar trend was found in terms of water absorption; lower values (13 to 32%) were found in *p*H_F_, *p*C_F_ and *p*WGB_F_ as compared to *p*CT. On the contrary, the cooking loss increased when pasta was fortified, being higher (up to 66%) when fermented ingredients were used. Hydration was also affected by the fortification; indeed, significantly higher values were found in fortified pasta especially when fermented doughs were used as ingredients. The highest value was reached in *p*WGB_F_.

Protein solubility in phosphate buffer was very low for all samples (< 2.91 ± 0.4 mg/g), while the addition of denaturing urea corresponded to a higher protein extraction (up to three-fold). The protein solubility in a buffer containing the disulfide reducing DTT was ca. two-fold lower compared to the phosphate buffer (data not shown), nevertheless, when both urea and DTT were used, the highest protein extraction was achieved. Overall, the protein solubility of fortified pasta was higher than the pasta containing only semolina. Moreover, the values of protein solubility were higher in fermented than the corresponding unfermented samples, probably due to the more intense proteolysis.

The hardness of fortified pasta samples was higher than *p*CT. However, when fermented doughs were used, lower values were found as compared to *p*H, *p*C and *p*WGB. Chewiness decreased with the fortification. However, when fermented doughs were used, it was higher in *p*C_F_ and *p*WGB_F_ as compared to *p*C and *p*WGB, respectively. The inclusion of wheat substitutes led to a decrease in the cohesiveness only when unfermented doughs were used. With the only exception of *p*H_F_, which showed lower value of chewiness, similar values were found between pasta containing fermented doughs and *p*CT.

Compared to *p*CT, lower lightness (L) and higher dE × ab values were found in all fortified pasta samples (Table 4). The highest “a” value, index for greenness (−)/redness (+) was observed for *p*WGB and *p*WGB_F_ (Table 4).

### 3.5. Nutritional Properties of Pasta

As expected, the fortification with H, C and WGB improved the content of protein and total dietary fibers (Table 5). A fiber concentration higher than 6% was obtained with the fortification. Nevertheless, fortification also increased the content of the ANF, although the use of fermented doughs corresponded to lower concentration than corresponding unfermented controls. Overall, ca. ten-fold decreases in phytic acid and raffinose were observed in pasta with fermented doughs (Table 5). *p*C_F_ and *p*H_F_ contained the lowest amount of phytic acid and raffinose, respectively. Condensed tannins were from 23% to 59% lower in *p*H_F_, *p*C_F_ and *p*WGB_F_ as compared to the corresponding pasta with unfermented doughs.

Pasta samples containing fermented doughs were characterized by lower values of HI (up to 79%) as compared to the corresponding unfermented ones, except for *p*H_F_. The use of unfermented and fermented milling by-products led to the lowest values of HI (60.62 and 42.4, respectively). The *p*GI of unfermented and fermented pasta ranged from 72.99 to 81.27 and from 62.98 to 79.74, respectively. The lowest value was found in *p*WGB_F_. A similar trend was found in terms of the IVPD. Increases from 22% to 45% were found in fortified pasta as compared to *p*CT. Values 43–64% higher than *p*CT were found in *p*H_F_, *p*C_F_ and *p*WGB_F_ (Table 5). The fermentation led to pasta having an IVPD from 10% to 22% higher than the corresponding pH, *p*C and *p*WGB.

### 3.6. Sensory Analysis

**Figure 2:**
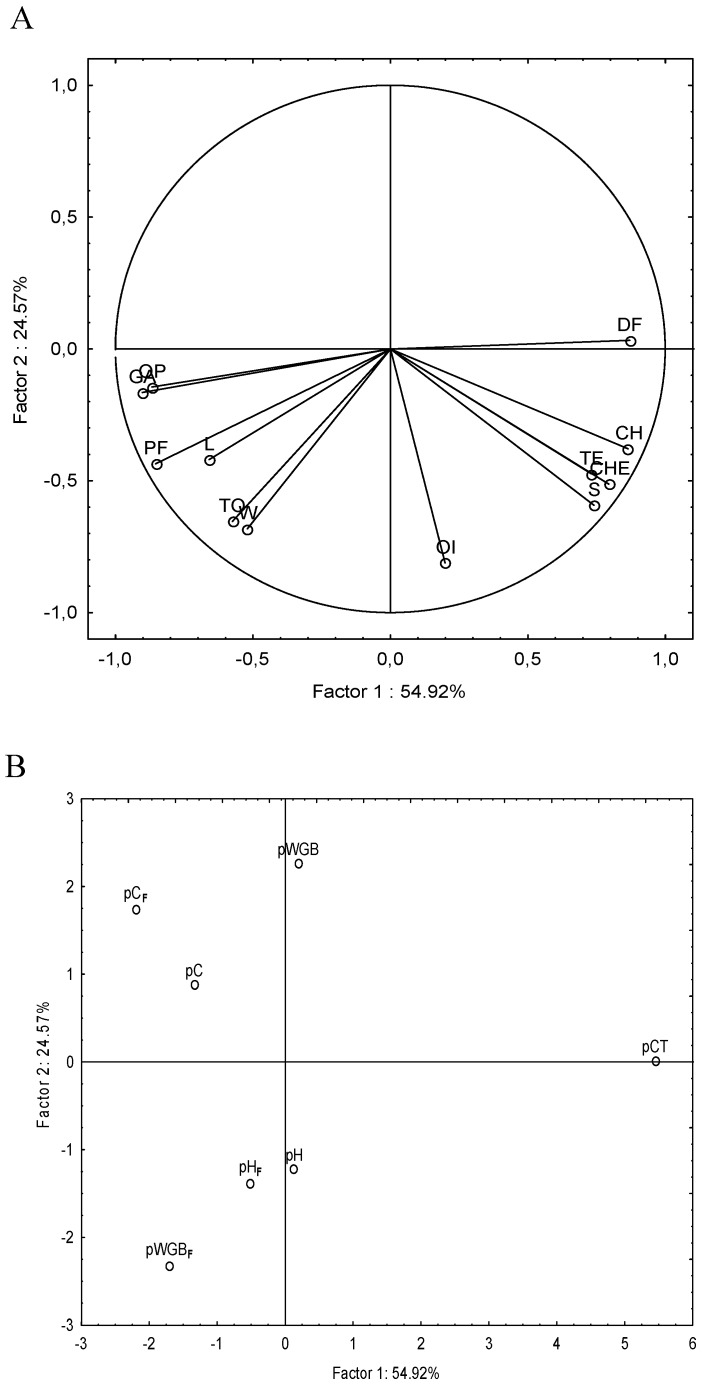
Loading plot (**A**) and score plot (**B**) of first and second principal components after principal component analysis (PCA) based on based on sensory analysis of pasta: *p*H, pasta containing 11% (*wt*/*wt*; d.m.) unfermented hemp dough; *p*H_F_, pasta containing 11% (*wt*/*wt*; d.m.) fermented hemp dough; *p*C, pasta containing 11% (*wt*/*wt*; d.m.) unfermented chickpea dough; *p*C_F_, pasta containing 11% (*wt*/*wt*; d.m.) fermented chickpea dough; *p*WGB, pasta containing 17% (*wt*/*wt*; d.m.) unfermented wheat germ and bran (1:4) dough; *p*WGB_F_, pasta containing 17% (*wt*/*wt*; d.m.) fermented wheat germ and bran (1:4) dough; *p*CT, pasta made only with semolina and water. The data are the means of three independent experiments ± standard deviations (*n* = 3). The attributes used in sensory analysis were: GA, general acceptability; OI, Odor Intensity; OP, Odor pungent; CH, Color Heterogeneity; S, Sapidity; PF, Pungent Flavor; DF, Delicate Flavor; TE, Texture; CHE, Chewability; ST, Stacking; L, Legume; W, Whole; TO, Toasted.

Pasta was subjected to sensory analysis and the results are summarized in Figure 2. The PCA, representing 79.49% of the total variance of the data, showed that pasta samples are scattered in different parts of the plane according to the raw materials used for the production. All fortified samples were in a different part of the plane as compared to the control *p*CT, thus confirming the strong influence of the fortification on the sensory profile of pasta. Moreover, among fortified samples, the fermentation seemed to strongly affect the sensory profile of pasta only when milling by-products were used. Indeed, *p*WBG_F_ and *p*WBG were scattered in different part of the plane. The former was characterized by a greater intensity of pungent odor and flavor and note of whole grains as compared to the corresponding *p*WBG. Only slight differences were found between *p*C and *p*C_F_ and *p*H and *p*H_F_, respectively. *p*C_F_ differentiated from the former due to the most intense legume note.

## 4. Discussion

The need for a diversified, balanced and healthy diet and the continued emphasis on the importance of dietary proteins and fibers are pressuring food companies and researchers to develop new products. Pasta is an important staple food; compared to other wheat-based foods, it is characterized by a lower glycemic index (GI), and it has been identified as a suitable carrier of bioactive compounds in daily diet [4,5,20]. Novel pasta recipes including the replacement of wheat flour with alternative flours, as well as the inclusion of pre-fermented ingredients have been recently proposed [20].

Here, hemp, chickpea and milling by-products were used to fortify semolina pasta. Aiming at improving the protein bio-accessibility and digestibility of the non-wheat flours and milling by-products before pasta making, treatments with food–grade enzymes and fermentation were investigated. Proteases from *A. niger*, commercial xylanase (Depol 761P) and LAB (*L. plantarum* LB1 and *L. rossiae* LB5) have been used as pre-treatment of the raw materials. The first selection of the more suitable bioprocess option was carried out by the evaluation of the peptide profiles and the TFAA concentration.

When hemp, chickpea and milling by-products were subjected to enzymatic treatments, a moderate increase in the peptides was observed as the consequence of the proteolysis of the native proteins (proteases) [38] and the release of soluble compounds from the fibrous cellular compartments (xylanase) [39]. On the contrary, fermentation led to a decrease in the peptides (up to ca. 70% lower) and a relevant increase in the TFAA (up to ca. 80% higher), suggesting an intense proteolysis operated by both endogenous and bacterial proteases and peptidases on proteins and their derivatives. It has largely been reported that the biological acidification operated by LAB lead to the activation of endogenous proteases which start the primary proteolysis where medium-sized polypeptides are released and subjected to LAB peptidase activities [40]. Based on these results, fermentation was chosen as the optimal bioprocessing option and its effects on hemp, chickpea, and milling by-products further investigated.

Aiming at investigating the suitability of hemp, chickpea and milling by-products as food ingredients, unfermented and fermented doughs were included in pasta formulation. In order to limit the weakening of the gluten network, the level of fortification was kept below 30% [7,41]. Nevertheless, the cooking performances and textural properties of fortified products were affected by the inclusion of the additional ingredients. A decrease in OCT and the increase in the cooking loss observed in fortified pasta might be due to the lower quality of the gluten network [20]. Overall, fortified pasta was characterized by values of hardness higher than control. However, the magnitude was lower when the fermentation was used as pre-treatment, regardless of the raw material. Data from panel test highlighted that the fortification affected the flavor of pasta. Indeed, *p*CT was characterized by the more intense delicate flavor, while legume, toasted and whole flavors were identified in fortified pasta, according to the raw materials used. The high intensity of pungent flavor and odor, due to the fermentation, also contributed to the differentiation among fortified pasta and *p*CT.

The fortification led to a pasta rich in fiber and protein, regardless of the fermentation process. Indeed, more than 13% of protein as well as ca. 6% (d.m) of fiber were achieved. According to EC Regulation [42] on nutrition and health claims on food products, experimental fortified pasta can be labelled as a “source of fiber” and a “source of protein”. Nevertheless, increases in the ANF, as compared to control pasta were found as a result of the fortification. The fermentation with selected LAB led to significant degradation (to traces) of the phytic acid, raffinose and condensed tannins as compared to the corresponding unfermented samples. Phytic acid (Myo-inositol 1,2,3,4,5,6 hexakis [dihydrogen phosphate]) is considered ANF due to the binding capacity towards essential dietary minerals, proteins and starch, thus reducing their bioavailability. The degradation of phytic acid during fermentation is achieved mainly through plant phytases [19] activated by LAB acidification. Moreover, a specific role of the organic acids on phytase activity has recently been proposed [43]. Cation chelation from organic acids may inhibit the aggregation of minerals and other molecules by phytic acid, thereby increasing their digestibility [43]. When present at high concentration, i.e., in legumes, raffinose is considered an ANF. However, LAB contribute to its enzymatic hydrolysis during fermentation [44], thus increasing product digestibility and reducing digestive discomfort [45]. The degradation of condensed tannins through LAB has already been proposed. It involves several enzymatic activities such as tannase, polyphenol oxidase and decarboxylase [46].

Beside the improvements in terms of ANF, an ca. 20% higher IVPD was found in fermented pasta samples as compared to the corresponding unfermented ones. Control pasta was characterized by an IVPD value 45% and 64% lower than the fortified samples (unfermented and fermented, respectively). Pasta containing non-wheat flours and milling by-products had a lower value of *p*GI compared to control, probably due to the higher concentration of dietary fibers and resistant starch, and a further decrease was found when the fermented flours were used. This effect could be attributed to biological acidification, which is among the main factors that decreases the starch hydrolysis rate and HI [36].

## 5. Conclusions

The study highlights the suitability of the fortification as a tool to improve the nutritional quality of pasta. Nevertheless, the pre-treatment of the non-wheat flours seems to be necessary to overcome the nutritional, structural and sensory drawback related to the use of such ingredients. Lactic acid bacteria fermentation has successfully been used to include hemp and chickpea flours and milling by-products in pasta making. LAB contributed to the increase in free amino acid content and decrease in phytic acid, raffinose and condensed tannins as compared to the corresponding unfermented doughs containing pasta. Moreover, fermentation improved protein digestibility and decreased the starch hydrolysis rate. Structural properties, cooking quality and sensory profiles were strongly affected by the fortification. Aiming at limiting the loss of rheological properties and cooking quality caused by the incorporation of non-wheat ingredients, further optimization of the technological processes may be needed.

## Figures and Tables

**Table 1:** Formulas for pasta fortified with hemp, chickpea, and wheat germ/bran unfermented and fermented doughs: *p*H, pasta containing 11% of unfermented hemp dough (*wt*/*wt*); *p*H_F_, pasta containing 11% of fermented hemp dough (*wt*/*wt*); *p*C, pasta containing 11% of unfermented chickpea dough; *p*C_F_ (*wt*/*wt*), pasta containing 11% of fermented chickpea dough (*wt*/*wt*); *p*WGB, pasta containing 17% of unfermented wheat germ and bran (1:4) dough (*wt*/*wt*); *p*WGB_F_, pasta containing 17% of fermented wheat germ and bran (1:4) dough (*wt*/*wt*); *p*CT, pasta made with durum wheat semolina.

	*p*H	*p*C	*p*WGB	*p*H_F_	*p*C_F_	*p*WGB_F_	*p*CT
Semolina (%)	70.2	70.2	70.2	70.2	70.2	70.2	77
Water (%)	19.8	19.8	12.8	19.8	19.8	12.8	23
Fermented dough (%) **^1^**	-	-	-	11	11	17	-
Unfermented dough (%) **^1^**	11	11	17	-	-	-	-

^1^ Having DY of 160, 11% of hemp (H and H_F_) and chickpea (C and C_F_) doughs contained ca. 6.8% of solids and 4.2% of water. While, 17% of wheat germ/bran doughs (WGB and WGB_F_) having dough yield (DY) of 250 contained ca. 6.8% dry matter and 10.2% of water. Consequently, all the fortified pasta samples contained ca. 7% of non-wheat flours/milling by-products.

**Table 2:** Proximate composition and microbiological characterization of hemp and chickpea flours and wheat germ/bran mixture.

	Hemp	Chickpea	Wheat germ:bran (1:4)
Protein (%)	36.9 ± 0.1 ^a^	25.2 ± 0.4 ^b^	17.8 ± 0.5 ^c^
Fat (%)	11.9 ± 0.2 ^a^	3.9 ± 0.1 ^c^	8.6 ± 0.7 ^b^
Available carbohydrates (%)	4.2 ± 0.1	48.9 ± 1.9	34.2 ± 2.1
Total dietary fiber (%)	39.7 ± 0.1 ^a^	24.3 ± 0.3 ^b^	37.8 ± 1.1 ^a^
Ash (%)	7.6 ± 0.2 ^a^	2.9 ± 0.9 ^b^	2.0 ± 0.4 ^b^

Data are expressed % of dry matter. ^a–c^ Values in the same row with different superscript letters differ significantly (*p* < 0.05).

**Table 3:** Chemical characterization of unfermented/fermented hemp, chickpea and wheat germ/bran doughs. H, dough made with hemp flour; H_F_, fermented dough made with hemp flour; C, dough made with chickpea flour; C_F_, fermented dough made with chickpea flour; WGB, dough made with a mixture of wheat germ and bran (1:4); WGB_F_, fermented dough made with a mixture of wheat germ and bran (1:4).

	H	C	WGB	H_F_	C_F_	WGB_F_
pH	6.4 ± 0.2 ^a^	6.1 ± 0.1 ^a^	6.3 ± 0.5 ^a^	4.5 ± 0.3 ^b,c^	4.4 ± 0.2 ^c^	4.9 ± 0.2 ^b^
Total titratable acidity (TTA) (mL NaOH 0.1M)	4.2 ± 0.3 ^c^	4.1 ± 0.2 ^c^	4.0 ± 0.1 ^c^	35.8 ± 0.6 ^a^	28.6 ± 0.8 ^b^	29.1 ± 0.7 ^b^
Lactic acid (mmol/Kg)	3.3 ± 0.2 ^d^	4.1 ± 0.2 ^c^	4.1 ± 0.5 ^c^	214.9 ± 5.8 ^b^	180.7 ± 4.3 ^c^	246.4 ± 6.2 ^a^
Acetic acid (mmol/Kg)	1.8 ± 0.9 ^d^	1.1 ± 0.3 ^d^	2.6 ± 0.4 ^c^	43.9 ± 0.9 ^a^	42.7 ± 0.7 ^a^	31.6 ± 0.3 ^b^
Fermentation quotient (FQ)	1.8	3.7	1.6	4.7	4.3	7.8
Total free amino acid (TFAA) (mg/Kg)	990 ± 8 ^d^	1972 ± 12 ^c^	802 ± 5 ^e^	1799 ± 11 ^c^	2457 ± 16 ^a^	1929 ± 13 ^b^
Peptides (g/Kg)	0.62 ± 0.05 ^c^	2.60 ± 0.60 ^a^	0.81 ± 0.09 ^b^	0.49 ± 0.59 ^c^	0.75 ± 0.20 ^b,c^	0.77 ± 0.10 ^b,c^

Hemp and chickpea doughs had a DY of 160; wheat germ/bran dough had a DY of 250. The data are the means of three independent experiments ± standard deviations (*n* = 3). ^a–e^ Values in the same row with different superscript letters differ significantly (*p* < 0.05).

**Table 4:** Chemical, technological and structural properties of pasta fortified with unfermented/fermented hemp, chickpea and wheat germ/bran doughs: *p*H, pasta containing 11% (*wt*/*wt*; d.m.) unfermented hemp dough; *p*H_F_, pasta containing 11% (*wt*/*wt*; d.m.) fermented hemp dough; *p*C, pasta containing 11% (*wt*/*wt*; d.m.) unfermented chickpea dough; *p*C_F_, pasta containing 11% (*wt*/*wt*; d.m.) fermented chickpea dough; *p*WGB, pasta containing 17% (*wt*/*wt*; d.m.) unfermented wheat germ and bran (1:4) dough; *p*WGB_F_, pasta containing 17% (*wt*/*wt*; d.m.) fermented wheat germ and bran (1:4) dough; *p*CT, pasta made with durum wheat semolina.

	*p*H	*p*C	*p*WGB	*p*H_F_	*p*C_F_	*p*WGB_F_	*p*CT
**Chemical Properties**							
pH	6.41 ± 0.10 ^a^	6.37 ± 0.21 ^a^	6.42 ± 0.31 ^a^	5.12 ± 0.11 ^b^	5.16 ± 0.05 ^b^	5.06 ± 0.08 ^b^	6.47 ± 0.24 ^a^
TTA (mL NaOH 0.1M)	3.01 ± 0.13 ^c^	2.05 ± 0.41 ^d^	1.82 ± 0.09 ^d^	6.61 ± 1.01 ^a^	4.01 ± 0.61 ^b^	4.45 ± 0.91 ^b^	2.01 ± 0.37 ^d^
TFAA (mg/Kg)	238 ± 9 ^e^	400 ± 8 ^d^	203 ± 4 ^f^	1018 ± 11 ^b^	743 ± 7 ^c^	1529 ± 12 ^a^	102 ± 10 ^g^
**Technological Properties**							
Optimal Cooking Time (OCT) (min)	7.0 ± 0.2 ^c^	6.3 ± 0.1 ^d^	8.0 ± 0.2 ^b^	6.3 ± 0.1 ^d^	6.0 ± 0.1 ^e^	7.0 ± 0.1 ^c^	10.0 ± 0.1 ^a^
Water absorption (%)	94.4 ± 3.7 ^c^	105.4 ± 3.2 ^b^	110.6 ± 2.9 ^b^	105.3 ± 4.5 ^a^	99.6 ± 3.6 ^b^	98.4 ± 3.2 ^b^	125.1 ± 4.1 ^a^
Cooking loss (% of d.m.)	4.7 ± 0.1 ^d^	4.6 ± 0.1 ^d^	6.3 ± 0.1 ^b^	5.1 ± 0.1 ^c^	5 ± 0.2 ^c^	6.8 ± 0.1 ^a^	4.1 ± 0.1 ^e^
**S** **tructural Properties**							
Hardness (N)	7.99 ± 0.51 ^b^	7.34 ± 0.29 ^b,c^	11.6 ± 0.31 ^a^	7.13 ± 0.42 ^b^	4.60 ± 0.17 ^d^	6.98 ± 0.18 ^c^	4.24 ± 0.15 ^e^
Chewiness (N)	2.00 ± 0.10 ^b^	1.46 ± 0.05 ^c^	2.07 ± 0.08 ^b^	1.14 ± 0.08 ^d^	2.03 ± 0.15 ^b^	2.22 ± 0.13 ^b^	2.86 ± 0.07 ^a^
Cohesiveness	0.33 ± 0.03 ^b^	0.27 ± 0.04 ^b,c^	0.26 ± 0.02 ^c^	0.29 ± 0.02 ^b,c^	0.43 ± 0.06 ^a^	0.45 ± 0.04 ^a^	0.44 ± 0.04 ^a^
**Color Analysis**							
L	52.1 ± 2.4 ^c^	65.5 ± 4 ^a,b^	62.8 ± 3.1 ^b^	51.2 ± 1.9 ^c^	65.6 ± 4.3 ^a^	63.2 ± 2.1 ^b^	70.8 ± 1.2 ^a^
a	0.6 ± 0.1 ^c^	0.8 ± 0.2 ^c^	1.4 ± 0.4 ^a,b^	0.6 ± 0.4 ^b,c^	0.3 ± 0.2 ^c^	2.1 ± 0.6 ^a^	−0.6 ± 0.2 ^d^
b	9.3 ± 1.7 ^c^	18.5 ± 1.6 ^a^	19.9 ± 2.7 ^a^	9.6 ± 0.9 ^c^	22.9 ± 3.2 ^a^	14.7 ± 1.5 ^b^	17.7 ± 2.4 ^a^
ΔE	42.9 ± 1.2 ^a^	31.7 ± 4.2 ^b^	35.7 ± 1.9 ^b^	41.9 ± 1.1 ^a^	34.7 ± 0.6 ^b^	33.4 ± 2.4 ^b^	26.7 ± 2.3 ^c^

Hemp and chickpea doughs had a DY of 160; wheat germ/bran dough had a DY of 250. Consequently, all the fortified pasta samples contained 7% of non-wheat flours/milling by-products. The data are the means of three independent experiments ± standard deviations (*n* = 3). ^a–g^ Values in the same row with different superscript letters differ significantly (*p* < 0.05).

**Table 5:** Nutritional properties of pasta fortified with unfermented/fermented hemp, chickpea and wheat germ/bran doughs: *p*H, pasta containing 11% (*wt*/*wt*; d.m.) unfermented hemp dough; *p*H_F_, pasta containing 11% (*wt*/*wt*; d.m.) fermented hemp dough; *p*C, pasta containing 11% (*wt*/*wt*; d.m.) unfermented chickpea dough; *p*C_F_, pasta containing 11% (*wt*/*wt*; d.m.) fermented chickpea dough; *p*WGB, pasta containing 17% (*wt*/*wt*; d.m.) unfermented wheat germ and bran (1:4) dough; *p*WGB_F_, pasta containing 17% (*wt*/*wt*; d.m.) fermented wheat germ and bran (1:4) dough; *p*CT, pasta made with durum wheat semolina.

	*p*H	*p*C	*p*WGB	*p*H_F_	*p*C_F_	*p*WGB_F_	*p*CT
Protein (%)	14.77 ± 0.31 ^a^	13.50 ± 0.27 ^b^	13.32 ± 0.19 ^b^	14.54 ± 0.29 ^a^	12.95 ± 0.31	13.54 ± 0.24 ^b^	12.32 ± 0.28 ^c^
Fat (%)	2.61 ± 0.06 ^b^	3.94 ± 0.02 ^a^	2.01 ± 0.02 ^c^	2.53 ± 0.02 ^b^	4.02 ± 0.06 ^a^	1.99 ± 0.04	1.58 ± 0.01 ^d^
Available carbohydrates (%)	74.16 ± 1.02	75.48 ± 1.01	76.99 ± 0.97	73.98 ± 1.07	75.66 ± 0.98	77.11 ± 0.99	81.94 ± 0.67
Total dietary fibers (%)	6.71 ± 0.25 ^a^	5.89 ± 0.31 ^b^	6.10 ± 0.24 ^b^	6.84 ± 0.33 ^a^	6.10 ± 0.36 ^b^	5.96 ± 0.32 ^b^	3.05 ± 0.18 ^c^
Ash (%)	1.75 ± 0.10 ^a^	1.27 ± 0.13 ^b^	1.58 ± 0.12 ^a,b^	1.81 ± 0.13 ^a^	1.34 ± 0.17 ^b^	1.67 ± 0.19 ^a^	1.11 ± 0.10 ^b^
Phytic acid (g/100g)	1.01 ± 0.05 ^a^	0.35 ± 0.01 ^c^	0.12 ± 0.02 ^d^	0.62 ± 0.06 ^b^	0.03 ± 0.01 ^e^	0.12 ± 0.02 ^d^	n.d.
Raffinose (g/Kg)	0.11 ± 0.02 ^c^	0.33 ± 0.02 ^a^	0.17 ± 0.02 ^b^	0.05 ± 0.01 ^d^	0.11 ± 0.01 ^c^	0.17 ± 0.02 ^b^	n.d.
Condensed tannins (g/Kg)	3.43 ± 0.10 ^a^	4.08 ± 0.91 ^a^	2.21 ± 0.03 ^c^	2.61 ± 0.09 ^b^	1.96 ± 0.02 ^d^	0.90 ± 0.01 ^e^	n.d.
HI (%)	75.71 ±6.52 ^a^	70.73 ± 4.91 ^a^	60.62 ± 4.18 ^b^	72.92 ± 5.17 ^a^	55.61 ± 3.05 ^b^	42.4 ± 3.96 ^c^	75.9 ± 2.18 ^a^
IVPD (%)	58.5 ± 0.6 ^d^	65.61 ± 0.7 ^b^	55.01 ± 0.4 ^e^	64.63 ± 0.8 ^c^	73.80 ± 0.7 ^a^	66.83 ± 0.6 ^b^	45.1 ± 0.4 ^f^

Hemp and chickpea doughs had a DY of 160; wheat germ/bran dough had a DY of 250. Consequently, all the fortified pasta samples contained 7% of non-wheat flours/milling by-products. The data are the means of three independent experiments ± standard deviations (*n* = 3). ^a–f^ Values in the same row with different superscript letters differ significantly (*p* < 0.05). n.d.: not detected.

## Supplementary Materials

The following are available online at https://www.mdpi.com/2304-8158/8/12/604/s1. Table S1: Drying cycle used for making pasta; Table S2: List and definition of the attributes used for the sensory analysis made on pasta samples.
