# Supplementary material for: Use of Fermented Hemp, Chickpea and Milling By-Products to Improve the Nutritional Value of Semolina Pasta
Source: Foods. 2019 Nov 22;8(12):604. doi: 10.3390/foods8120604 (PMC6963495; doi:10.3390/foods8120604)
Supplement: Supplementary file 1 [file foods-08-00604-s001.pdf]

Supplementary Table S1. Drying cycle used for making pasta.

	VENTILATION			RECOVERY		
**CYCLE**	Temperature (°C)	U.R.%	Time (min)	Temperature (°C)	U.R.%	Time (min)
**1**	55	85	3	55	85	2
**2**	55	85	3	55	85	2
**3**	55	70	3	55	70	4
**4**	55	70	3	40	65	8
**5**	55	70	3	40	60	10
**6**	55	70	3	40	60	10
**7**	55	70	3	40	55	10
**8**	55	70	3	40	55	12
**9**	55	70	3	40	55	12
**10**	55	70	3	40	55	12
**11**	55	70	3	40	55	12
**12**	55	70	3	40	55	14
**13**	55	70	3	40	50	14
**14**	55	70	3	40	50	16
**15**	55	70	3	40	50	16
**16**	55	70	3	40	50	16
**17**	55	65	3	40	50	16
**18**	55	65	3	40	50	18
**19**	55	65	3	40	50	18
**20**	55	65	3	40	45	18
**21**	55	65	3	40	45	18
**22**	55	60	3	35	45	18
**23**	55	60	3	35	45	18
**24**	50	55	3	35	45	18
**25**	45	55	3	35	15	18
**26**	40	40	3	15	15	10
**27**	15	10	20	15	10	20
**28**	15	10	20	15	10	20

Supplementary Table S2. List and definition of the attributes used for the sensory analysis made on pasta samples.

Attributes	Abbreviation	Definition	Reference
Visual			
Color heterogeneity	CH	Heterogeneity of the pasta color evaluated after cooking	[7]
Texture	TE	Uniformity of the pasta texture at visual inspection	[49]
Stacking	ST	The degree of piece to piece adhesion	[50]
Odor			
Intensity	OI	Odor intensity of pasta	[7]
Pungent	OP	Odor pungent of pasta	
Flavour			
Pungent	PF	Flavour pungent of pasta	
Delicate	DF	Flavour delicate of pasta	
Legume	L	Flavor of lentils, beans, chickpeas, etc.	[51]
Whole	W	Flavor of whole-wheat flour, buckwheat	
Toasted	TO	Grilled and toasty flavor	
Sapidity	S	Sapidity taste	This study
Texture			
Chewability	CHE	The mouthfeel sensation of labored mastication due to sustained, elastic resistance from the sample.	[49]
General acceptability	GA	Overall appreciation	This study
